# EHMTI-0214. Headache syndromes - known to the unknown?

**DOI:** 10.1186/1129-2377-15-S1-C53

**Published:** 2014-09-18

**Authors:** K Rao, J Rekha, S Mohan

**Affiliations:** 1 Neurology, Gokulam Hospital, Salem, India; 2 Radiology, Gokulam Hospital, Salem, India; 3 Medicine, Gokulam Hospital, Salem, India

## Background

Headache, is a widely prevalent and a common clinical problem across the world.Primary headaches form the majority with the current prevalence of headache estimated to be 47%, globally (WHO). Migraine and TTH are the two commonest. Few headache disorders could raise controversy in diagnosis and treatment.Some headaches which appear like primary(migraine,TAC,TTH etc.)or the known secondary ones may have few peculiar/interesting findings contrary to the conventional descriptions & can pose challenges to the diagnosis,classification and also treatment.

## Aim

To critically analyse & try understanding the mechanisms in both primary and some of the secondary ones which are atypical in their presentation when critically looked at from a clinical, radiological and treatment point of view.

## Methods

25 cases of different types were meticulously looked into. Detailed clinical data,supportive investigations, radiological findings (MRI/CTangio) CSF/opthalmological examination findings (selected) treatment & clinical course on follow up.

## Results

25 cases (F:M-1.5:1) in the age group (16–69years). Various types of headaches with f/o migraine, TAC, CDH (IHS criteria) were noted. 4 of those had overlapping features. 2 had recent onset daily headache associated with binocular diplopia, s/o raised ICT. 1 had two episodes of headache with transient binocular diplopia. One had classical s/o TAC at presentation. Rest had strictly unilateral headache with subtle autonomic features mimicking TAC. Radiological abnormalities were seen in all excepting 2. Three cases mimicking BIH had normal MRV with abnormal CSF in 1 and all 3 had abnormal thyroid functions. Treatment was individualized and clinical response to drug regimens was varied.

## Conclusions

Headaches can be extremely challenging in their manifestations. Many, though appear to mimic the known ones, can have atypical findings when looked in critically. These could raise controversies in classification & treatment.

No conflict of interest.

